# The mitochondrial permeability transition pore: a mystery solved?

**DOI:** 10.3389/fphys.2013.00095

**Published:** 2013-05-10

**Authors:** Paolo Bernardi

**Affiliations:** Department of Biomedical Sciences, University of PadovaPadova, Italy

**Keywords:** mitochondria, permeability transition, calcium, F_O_F_1_ ATP synthase

## Abstract

The permeability transition (PT) denotes an increase of the mitochondrial inner membrane permeability to solutes with molecular masses up to about 1500 Da. It is presumed to be mediated by opening of a channel, the permeability transition pore (PTP), whose molecular nature remains a mystery. Here I briefly review the history of the PTP, discuss existing models, and present our new results indicating that reconstituted dimers of the F_O_F_1_ ATP synthase form a channel with properties identical to those of the mitochondrial megachannel (MMC), the electrophysiological equivalent of the PTP. Open questions remain, but there is now promise that the PTP can be studied by genetic methods to solve the large number of outstanding problems.

## The mitochondrial permeability transition

The permeability transition (PT) defines an increase of mitochondrial inner membrane permeability to ions and solutes with molecular masses up to about 1500 Da leading to matrix swelling. The occurrence of swelling in isolated mitochondria, its strict dependence on matrix Ca^2+^, stimulation by Pi and fatty acids, and inhibition by Mg^2+^ and adenine nucleotides has been recognized very early (Raaflaub, 1953a,b; Brenner-Holzach and Raaflaub, 1954; Hunter and Ford, 1955; Tapley, 1956; Lehninger, 1959; Lehninger and Remmert, 1959; Wojtczak and Lehninger, 1961; Zborowski and Wojtczak, 1963; Azzi and Azzone, 1965; Azzone and Azzi, 1965; Chappell and Crofts, 1965; Crofts and Chappell, 1965); and its detrimental effects on energy conservation have been described before the emergence of chemiosmotic concepts (Mitchell, 1961, 2011). As a result, the PT has initially been considered an *in vitro* artifact of questionable pathophysiological relevance, although a few Authors did recognize its potential importance in pathophysiology (Rasola and Bernardi, 2007).

Pfeiffer and Coworkers suggested that the permeabilizing effect of Ca^2+^ had a physiological role in steroidogenesis. These Authors showed that Ca^2+^ induces a “transformation” of adrenal cortex mitochondria allowing diffusion of extramitochondrial pyridine nucleotides through the otherwise impermeable inner membrane, and that NADPH entering in this way supports the 11-β hydroxylation of deoxycorticosterone (Pfeiffer and Tchen, 1973, 1975; Pfeiffer et al., 1976). These findings match those of Vinogradov et al., who demonstrated the Ca^2+^-dependent release of matrix pyridine nucleotides in liver mitochondria (Vinogradov et al., 1972). The term “permeability transition” was introduced by Haworth and Hunter, who thoroughly characterized its basic features in heart mitochondria. These Authors provided the key insight that the PT was due to reversible opening of a proteinaceous pore in the inner mitochondrial membrane, the permeability transition pore (PTP), whose physiological role remained undefined (Hunter et al., 1976; Hunter and Haworth, 1979a,b; Haworth and Hunter, 1979).

Studies on the PT were not too popular at this time, a possible consequence of the general acceptance of the chemiosmotic hypothesis which had just been fully recognized with the award of the Nobel Prize to Peter Mitchell in 1978. As noted earlier (Bernardi, 1999) studies of mitochondrial ion transport were indeed carried out in the same laboratories involved in clarifying the mechanisms of energy conservation, and they tended to become tests of the predictions of the chemiosmotic theory. In retrospect it is not too surprising that the existence of a large pore in the inner membrane appeared to contradict the basic tenets of chemiosmosis. As a result, and with very few exceptions (see e.g., Beatrice et al., 1980; Coelho and Vercesi, 1980; Bernardi and Pietrobon, 1982; Lê-Quôc and Lê-Quôc, 1982, 1985; Siliprandi et al., 1983; Vercesi, 1984; Riley and Pfeiffer, 1985; Al Nasser and Crompton, 1986; Sokolove and Shinaberry, 1988) research in this area did not enjoy much popularity.

The discovery that in mammalian mitochondria the PT can be inhibited by submicromolar concentrations of cyclosporin (Cs) A (Fournier et al., 1987; Crompton et al., 1988; Broekemeier et al., 1989; Davidson and Halestrap, 1990) changed the field substantially. CsA inhibits the PTP after binding to matrix cyclophilin (CyP)D, a peptidyl-prolyl *cis-trans* isomerase (PPIase) whose enzymatic activity is blocked by CsA (Fischer et al., 1989; Takahashi et al., 1989) in the same range of concentrations inhibiting the pore (Connern and Halestrap, 1992, 1994; Nicolli et al., 1996; Woodfield et al., 1997). A second fundamental finding was the demonstration that mitochondria possess ion channels that can be studied by electrophysiology (Sorgato et al., 1987). This seminal study was soon followed by the demonstration that the inner mitochondrial membrane is endowed with a high-conductance (≈1–1.3 nS) channel, the “mitochondrial megachannel” (MMC) (Kinnally et al., 1989; Petronilli et al., 1989). The MMC is inhibited by CsA (Szabó and Zoratti, 1991) and possesses all the key regulatory features of the PTP (Bernardi et al., 1992; Szabó et al., 1992) leaving little doubt that the PTP and the MMC are two aspects of the same molecular entity (Szabó and Zoratti, 1992). Electrophysiology has greatly contributed to our understanding of the MMC–PTP, and to acceptance of the pore theory of the PT (Zoratti et al., 2005).

Involvement of the PT in cell death was hypothesized 25 years ago (Crompton and Costi, 1988). Early support was obtained in hepatocytes subjected to oxidative stress (Broekemeier et al., 1992; Imberti et al., 1992), anoxia (Pastorino et al., 1993), or treatment with ATP (Zoeteweij et al., 1993); and in cardiomyocytes (Duchen et al., 1993) and isolated hearts (Griffiths and Halestrap, 1995) exposed to ischemia followed by reperfusion. The exponential increase in experimental papers dealing with the PT as an effector mechanism of cell death, however, only followed the demonstration that in the course of apoptosis cytochrome c is released into the cytosol (Liu et al., 1996) together with apoptosis-inducing factor (Susin et al., 1996) and a set of other proteins involved in the effector phase of apoptosis (Du et al., 2000; Ekert et al., 2000; Hegde et al., 2001; Li et al., 2001).

The molecular basis of the PT is still the matter of conjectures (Siemen and Ziemer, 2013). The various models and working hypotheses proposed over the years had to cope with the lack of selectivity for the permeating species, the strict requirement for matrix Ca^2+^, which has recently been established also for yeast mitochondria (Yamada et al., 2009), the stimulation by oxidants, the existence of a vast number of “inducers” without common structural features, and of a more limited but still substantial number of inhibitors (Gunter and Pfeiffer, 1990).

## Many suspects, no culprits

### Adenine nucleotide translocator, VDAC, and contact sites

Inhibition of mitochondrial swelling by adenine nucleotides has long been appreciated (Raaflaub, 1953b; Brenner-Holzach and Raaflaub, 1954), but the idea that the adenine nucleotide translocator (ANT) is causally involved in the PT probably originates from the finding that Ca^2+^-dependent membrane permeabilization is affected by the ANT inhibitors atractylate and bongkrekate, atractylate being a PT inducer and bongkrekate a PT inhibitor (Hunter and Haworth, 1979a). While early studies concluded (correctly, as it turns out) that the PT is an inner membrane event, the outer membrane was called into the picture after the work of Brdiczka and coworkers on inner-outer membrane contact sites, i.e., specialized structures where the two membranes form close contacts mediated by protein–protein interactions (Kottke et al., 1988). The contact sites were proposed to facilitate channeling of adenine nucleotides to and from mitochondria, and to comprise hexokinase on the cytosolic surface and VDAC within the outer membrane, creatine kinase and nucleoside diphosphate kinase (in tissues that express these proteins), and ANT in the inner membrane (Adams et al., 1989; Bucheler et al., 1991). The connection with the PTP was made in 1996, when the same laboratory found that hexokinase-enriched fractions formed channels with the conductance expected of the PTP, and conferred permeability properties to liposomes that could be inhibited by N-methylVal-4-cyclosporin (Beutner et al., 1996). Unlike the case of MMC, however, currents were inhibited rather than induced by atractylate; the active fractions were not enriched in VDAC or ANT; and the preparation contained a very large number of proteins (Beutner et al., 1996) making assignment of the channel activity to a specific species quite problematic. The same hexokinase-enriched fractions were used to study the interactions with proteins of the Bcl-2 family (Marzo et al., 1998). The hexokinase/VDAC/ANT model of the PTP was extended to include outer membrane TSPO [formerly known as peripheral benzodiazepine (Bz) receptor] and Bcl-2 family members (Zamzami and Kroemer, 2001). No further progress was made in the purification of the component(s) mediating channel activity and membrane permeabilization, yet this model became widely accepted as the “structure” of the PTP.

### What did we learn from genetic inactivation of putative PTP components

Conclusive evidence that the ANT is not essential for PTP formation comes from analysis of mitochondria lacking all ANT isoforms, which revealed that a Ca^2+^-dependent PT took place (Kokoszka et al., 2004). The PT could be inhibited by CsA and triggered by thiol oxidants, demonstrating that the ANT is neither the obligatory binding partner of CyPD nor the site of action of oxidants (Kokoszka et al., 2004) as suggested earlier (Costantini et al., 1998). In addition, hepatocytes prepared from control and ANT-deficient livers showed identical responses to activation of receptor-mediated apoptotic pathways initiated by TNFα and Fas (Kokoszka et al., 2004). It has been argued that a low, undetectable level of ANT expression could explain the PT observed in ANT-null mitochondria (Halestrap, 2004). I believe that this possibility is quite remote because the PTP of mitochondria lacking ANT was insensitive to opening by atractylate and to closure by ADP (Kokoszka et al., 2004). As noted elsewhere (Bernardi et al., 2006) it is very difficult to envisage how any ANT molecules present in mutant mitochondria would promote a CsA-sensitive PT and yet not respond to atractylate and ADP.

A detailed comparison of the PT in mitochondria from wild-type and *Vdac1^−/−^* mice demonstrated that VDAC1 is fully dispensable for PTP opening and regulation (Krauskopf et al., 2006). Furthermore, the PT of mitochondria prepared from *Vdac3^−/−^* and *Vdac1^−/−^Vdac3^−/−^* mice, or from fibroblasts lacking all three VDAC isoforms, was identical to that of strain-matched wild-type mitochondria (Baines et al., 2007). Taken together, these results clearly indicate that VDACs are not components of the PTP although, as discussed later in the review, the outer membrane appears to participate in PTP regulation (Sinha Roy et al., 2009; Šileikyte et al., 2011).

TSPO is an 18 kDa highly hydrophobic protein, involved in cholesterol and protophorphyrin transport, which is located in the outer mitochondrial membrane (Batarseh and Papadopoulos, 2010). It was initially identified as a binding site for Bz in tissues that lack GABA receptors (Anholt et al., 1985, 1986). TSPO binds with nM affinity a variety of ligands, notably the Bz Ro5-4864 and the isoquinoline carboxamide PK11195 (Verma et al., 1987) as well as protoporphyrin IX, a potent inducer of the PTP (Pastorino et al., 1994). Treatment with TSPO ligands does affect the channel activity of the MMC–PTP in the proper concentration range (Kinnally et al., 1993) but TSPO is not the only mitochondrial protein binding these compounds, which have been shown to interact with, and inhibit, the F_O_F_1_ ATP synthase (Cleary et al., 2007). The latter finding acquires a particular meaning in the light of our recent demonstration that F_O_F_1_ ATP synthase dimers form channels indistinguishable from the PTP that can be activated by Bz-423 (Giorgio et al., 2013). Perhaps not surprisingly then, the PTP of mitochondria from mice where expression of TSPO had been conditionally inactivated could still be sensitized to Ca^2+^ by TSPO ligands, and had general features indistinguishable from those of mitochondria isolated from mice with wild-type genotype (Šileikytė, Forte et al., unpublished observations).

In summary, what we learned from gene inactivation studies is that none of the channel-forming proteins suggested to take part in PTP formation is necessary for its occurrence.

### Alternative models for PTP formation

The Halestrap laboratory has proposed a new model for the PTP that envisions the formation of a pore by the Pi carrier (Leung et al., 2008). PTP formation would be favored by Ca^2+^ and CyPD binding, and the open probability would be increased by formation of a heterodimer with the ANT (Leung and Halestrap, 2008). This model still needs to be tested by reconstitution and by genetic means, but it should be noted that patch-clamp experiments with the functionally active mitochondrial Pi carrier revealed an anion-selective channel function with a mean conductance as low as 40 pS (the typical conductance of the PTP is 1.0−1.3 nS, and the channel is not ion-selective). Furthermore, the current was decreased to 25 pS by both Ca^2+^ (which activates the PTP) and Mg^2+^ (which inhibits it). Finally, and at striking variance with the pore, channel activity was inhibited by Pi and unaffected by ADP (Herick et al., 1997). These features make the Pi carrier a very unlikely candidate as a PTP component.

A radically different model of the PTP has been proposed in which the pore does not form from a specific protein but rather from aggregation of misfolded integral membrane proteins damaged by oxidant and other stresses (Kowaltowski et al., 2001; He and Lemasters, 2002). In the hypothesis of He and Lemasters conductance through misfolded protein clusters would be normally blocked by chaperone-like proteins (including CyPD) and modulated by Ca^2+^ and CsA. Opening of “unregulated” pores would occur when protein clusters exceed available chaperones (He and Lemasters, 2002). This model fails to account for the absolute Ca^2+^ requirement of PTP, and its regulation by voltage (Bernardi, 1992), matrix pH (Nicolli et al., 1993), and adenine nucleotides, all effects that are not easy to reconcile with a permeability pathway created by a heterogeneous set of denatured proteins.

It should be mentioned that Ca^2+^-dependent, CsA-insensitive PT-like channel activities have been described that are formed or activated by 3-hydroxybutyrate/polyphosphate (Pavlov et al., 2005) and by fatty acids (Mironova et al., 2001; Sultan and Sokolove, 2001). The latter observation links to early work demonstrating that fatty acids are potent inducers of mitochondrial swelling (Lehninger and Remmert, 1959; Wojtczak and Lehninger, 1961), which was later recognized to be due to PTP opening (Scorrano et al., 2001; Bernardi et al., 2002).

## Cyclophilin D and F_o_F_1_ ATP synthase

CyPs are ubiquitous, conserved proteins possessing PPIase activity (Fischer et al., 1989; Takahashi et al., 1989) sharing a common domain of about 109 amino acids, the CyP-like domain (Wang and Heitman, 2005). In man 16 unique CyPs have been found, the prototype being cytosolic CyPA (Wang and Heitman, 2005). After binding CsA the PPIase activity is inhibited (Borel et al., 1977) and the CsA/CyPA complex binds to and inhibits the cytosolic phosphatase calcineurin (Liu et al., 1991), causing immunosuppression (Clipstone and Crabtree, 1992; Walsh et al., 1992).

CyPD is the mitochondrial CyP isoform in mammals (Connern and Halestrap, 1992; Nicolli et al., 1996; Woodfield et al., 1997), see Giorgio et al. (2010) for a review. Much on its role in PTP regulation has been learned by genetic ablation of the *Ppif* gene (which encodes for CyPD). CyPD is the mitochondrial receptor for CsA and modulates the PTP but is not a structural pore component (Baines et al., 2005; Basso et al., 2005; Nakagawa et al., 2005; Schinzel et al., 2005). As discussed elsewhere (Bernardi et al., 2006), the effect of CsA on the PTP is best described as “desensitization” in the sense that the PTP becomes more resistant to opening by Ca^2+^ and Pi. It should be stressed that pore opening readily takes place for Ca^2+^-Pi loads that are about twice those required in wild-type mitochondria. This indicates that *Ppif^−/−^ (CyPD-null) mitochondria are not null for the PTP.* This consideration is important for the interpretation of results obtained with CsA because (similarly to the absence of CyPD) CsA can desensitize but not block the PTP; thus, lack of sensitivity to CsA does not necessarily mean that the PTP is not involved in the event being studied. Furthermore, expression of CyPD is modulated, e.g., by muscle denervation (Csukly et al., 2006), and only CyPD-expressing mitochondria respond to CsA (Li et al., 2012). No endogenous ligands of CyPD mimicking the effects of CsA are known, but other PTP (de)sensitizers might act by favoring CyPD association or dissociation, as documented for acidic pH and increasing ionic strength (Nicolli et al., 1996). Furthermore, CyPD phosphorylation (Rasola et al., 2010), acetylation (Shulga and Pastorino, 2010), and nitrosylation (Kohr et al., 2011; Nguyen et al., 2011) affect the propensity of the PTP to open (Rasola and Bernardi, 2011). Not surprisingly CyPD may interact with many proteins including Hsp90 and its related molecule TRAP-1 (Kang et al., 2007); Bcl-2 (Eliseev et al., 2009); ERK-2/GSK-3 (Rasola et al., 2010); possibly p53 (Vaseva et al., 2012), but see (Karch and Molkentin, 2012); and the F_O_F_1_ ATP synthase (Giorgio et al., 2009). The latter interaction turned out to be the key for identification of the PTP.

**Figure 1:**
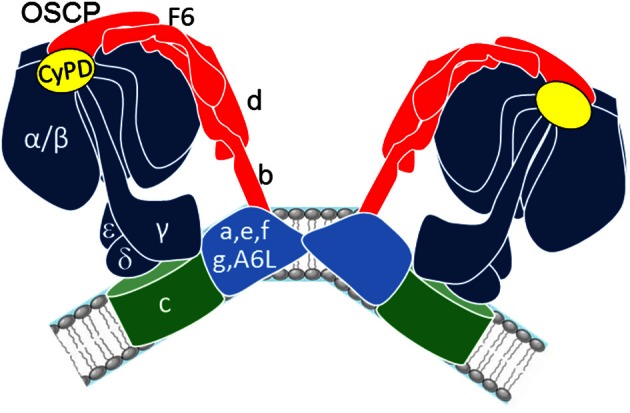
**Schematic representation of F_O_F_1_ ATP synthase dimers.** F_1_ (dark blue), F_O_ (green and light blue), and stalk subunits (red) are illustrated based on recent structural studies (Strauss et al., 2008; Baker et al., 2012; Davies et al., 2012).

The F_O_F_1_ ATP synthase (or complex V) is the rotary enzyme that synthesizes the vast majority of ATP in respiring cells (Rees et al., 2009). It is formed by the catalytic F_1_, the membrane-bound proton-translocating F_O_, and the lateral stalk linking F_1_ and F_O_ (Strauss et al., 2008; Thomas et al., 2008; Rees et al., 2009; Baker et al., 2012; Davies et al., 2012) (Figure 1). The lateral stalk acts as a stator preventing the α_3_β_3_ subcomplex of F_1_ from rotating with subunits γ, δ, and ε and the ring of F_O_-subunits c (Rees et al., 2009). The F_O_F_1_ ATP synthase complex associates to form dimers, which are considered to be the “building blocks” of long rows of oligomers that promote cristae formation and may optimize the enzyme's catalytic performance (Campanella et al., 2008; Strauss et al., 2008; Thomas et al., 2008; Rees et al., 2009; Baker et al., 2012; Davies et al., 2012). The angle formed by the dimers seems to display a large variability, and dimers are believed to represent the physiological unit of complex V in the inner membrane (Thomas et al., 2008).

We found that CyPD binds the lateral stalk of complex V in an apparent ratio of 1:1:1:1 with the OSCP, b and d subunits (Giorgio et al., 2009). CyPD binding requires Pi and results in partial inhibition of ATP synthase activity, while CsA displaces CyPD resulting in enzyme reactivation (Giorgio et al., 2009). The stimulatory effect of CsA on enzyme catalysis is lost in *Ppif*^−/−^ mitochondria, proving that it is mediated by CyPD, while assembly of the ATP synthase is not affected by CyPD ablation (Giorgio et al., 2009). In spite of the striking analogies with PTP regulation (Pi dependence, CsA sensitivity) whether the interactions of CyPD with complex V are relevant for the PT remained unknown until very recently.

## Dimers of F_o_F_1_ ATP synthase form channels indistinguishable from the MMC–PTP

We have defined the binding site of CyPD on ATP synthase to the OSCP subunit, and shown that the interaction is electrostatic in nature. Modeling of surface potentials and isopotential curves suggested that CyPD interacts with OSCP in a region overlapping with helices 3 and 4, and this turned out to be a key insight (Giorgio et al., 2013). Indeed, this is also the binding site of Bz-423, a well-characterized inhibitor of the F_O_F_1_ ATP synthase (Johnson et al., 2005; Stelzer et al., 2010). If the CyPD interactions are important for the PT, it was logical to expect that Bz-423 should act as a PTP inducer, and this was the case. Bz-423 sensitized the PTP to Ca^2+^, and the inducing effect was blunted by Pi at concentrations that increase CyPD binding to OSCP (Giorgio et al., 2013). Consistent with an involvement of complex V in PTP formation, decreased levels of OSCP did affect the Ca^2+^ dependence of the pore. Indeed, mitochondria took up Ca^2+^ normally when ATP was used as the energy source, but the threshold Ca^2+^ load required for PTP opening was halved, which is the first molecular evidence that OSCP affects the probability of pore opening. Consistent with a key role of the ATP synthase, the Ca^2+^ affinity of the PTP was also affected by enzyme catalysis in the sense that ATP-hydrolyzing mitochondria required twice the Ca^2+^ load of ATP-synthesizing organelles, an effect as large as that of CsA (Giorgio et al., 2013).

Direct evidence that the PTP forms from the F_O_F_1_ ATP synthase was obtained by incorporating dimers purified by blue native electrophoresis into azolectin bilayers, and by measuring the passage of currents after application of a voltage difference. Addition of Bz-423 in the presence of Ca^2+^ triggered opening of high-conductance channels that were blocked by Mg^2+^/ADP and by the ATP synthase inhibitor AMP-PNP (γ-imino ATP, a non hydrolyzable ATP analog). Monomers of ATP synthase were devoid of channel activity in spite of the same overall subunit composition as the dimers (Tomasetig et al., 2002; Giorgio et al., 2013). The characteristics of the reconstituted pore closely matched those of MMC–PTP (Petronilli et al., 1989; Szabó and Zoratti, 1991; Szabó et al., 1992). Maximal chord conductance was between 1.0 and 1.3 nS in symmetrical KCl 150 mM, and various subconductance states were observed. Like the MMC of *Ppif*^−/−^ mitochondria (De Marchi et al., 2006), and in keeping with the lack of CyPD in the preparations, the channel was insensitive to CsA. Ca^2+^ and Pi, which sensitize the PTP even in the absence of CyPD, sufficed to induce PTP currents when dimers were prepared in the presence of 10 mM Pi. Channel openings could not be elicited by atractyloside and, once elicited by Bz-423, were still observed in the presence of bongkrekic acid. These findings are consistent with the absence of ANT in the preparations, which also totally lacked VDAC (Giorgio et al., 2013).

Bz-423 was characterized as an apoptosis-inducing agent acting through mitochondria (Blatt et al., 2002) and OSCP was identified as its target through the unbiased screening of a human phage display library (Johnson et al., 2005). The selectivity of action of Bz-423 on OSCP, its ability to trigger channel activity of ATP synthase dimers, and the lack of activity of monomer preparations strongly argues against the possibility that the currents observed by Giorgio et al. (2013) are due to unidentified contaminating proteins. The PTP-inducing effect of Bz-423 and CyPD (which both act through OSCP on top of the lateral stalk in the matrix) must be indirect, as it affects the permeability properties of the inner membrane. We assume that the PTP forms at the membrane interface between two adjacent F_O_ sectors, which could also accommodate the effects of fatty acids (Lehninger and Remmert, 1959; Wojtczak and Lehninger, 1961; Bernardi et al., 2002). Matrix Ca^2+^ has an essential permissive role in PTP formation, and we have suggested that accessibility of Ca^2+^ to the metal binding sites of the catalytic F_1_ sector is influenced by OSCP. This subunit could indeed affect the affinity of the metal binding sites of ATP synthase and thus determine the ease with which matrix Ca^2+^ can replace Mg^2+^ causing PTP opening. Our working hypothesis is that OSCP as such is a “negative” modulator, whose effect can be counteracted by binding of the “positive” effector CyPD (which indeed increases the apparent Ca^2+^ affinity of the PTP). Removal of OSCP, or CyPD binding to OSCP, would induce similar conformational effects leading to increased probability of PTP opening, a working hypothesis that awaits experimental verification.

**Figure 2:**
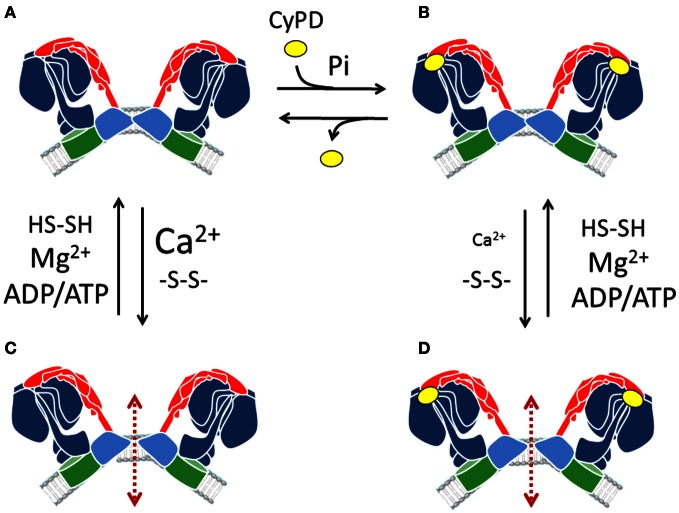
**Hypothetical transition of F_O_F_1_ ATP synthase dimers to form the PTP.** ATP synthase dimers **(A)** can undergo PTP formation when Ca^2+^ rather than Mg^2+^ is bound, possibly at the catalytic sites, in a reversible process favored by thiol oxidation **(C)**. Binding of CyPD, which is favored by Pi **(B)** would increase the accessibility of the metal binding sites, allowing PTP formation at lower Ca^2+^ concentrations (as depicted here by a smaller face type) **(D)**. Adenine nucleotides counteract PTP formation in synergy with Mg^2+^. Red arrows denote the hypothetical pathway for solute diffusion between two F_O_ subunits.

A cartoon with the hypothetical events leading to PTP formation from complex V dimers is presented in Figure 2. In the “coupled” condition the dimers bind Mg^2+^-ADP/ATP, and the concentration of free Ca^2+^ (and Pi, not shown in the Figure) is not high enough for the PT to occur (panel **A**). Binding of CyPD, which is favored by Pi (Giorgio et al., 2009), would cause a conformational change affecting accessibility of the metal binding sites. This in turn would decrease the matrix Ca^2+^ load necessary to trigger the PT (panels **B,D**). The transition would essentially depend on replacement of Mg^2+^ with Ca^2+^, which in the absence of CyPD binding would require higher matrix Ca^2+^ loads (panel **C**). Accessibility of the metal binding site (and probability of occurrence of the PT at a given Ca^2+^ load) would be increased by thiol oxidation and counteracted by thiol reduction. Ion and solute permeation would then occur at the interface between the c rings (panels **C,D**). The PT can be fully reverted by Ca^2+^ chelation with EGTA (Petronilli et al., 1994), which would restore Mg^2+^ binding and allow the dimer to recover the coupled structure.

## A mystery solved, more mysteries to solve

### Role of outer membrane

It can no longer be questioned that the PT is an inner membrane event since (1) complex V dimers suffice to induce MMC activity and (2) Ca^2+^-dependent PT occurs in mitoplasts, i.e., mitochondria stripped of the outer membrane (Šileikyte et al., 2011). These findings should not be taken to imply that the outer membrane does not play a role in PTP modulation, which is in fact supported by a large number of studies. An early indication was provided by Lê-Quôc and Lê-Quôc, who showed that induction of the PTP by substituted maleimides requires the outer membrane (Lê-Quôc and Lê-Quôc, 1985). We have fully confirmed this “sensitizing” role of the outer membrane by an independent approach, i.e., irradiation of mitochondria with visible light after treatment with hematoporphyrin, a strategy that allows production of singlet oxygen leading to PTP inactivation or reactivation depending on the light dose (Ricchelli et al., 2011). Low light doses inactivate the PTP through degradation of hystidyl residues that prevent cysteine oxidation on the matrix side (Salet et al., 1997), while higher light doses activate the PTP through cysteine oxidation at the outer membrane (Moreno et al., 2001; Petronilli et al., 2009). Mitoplasts are completely resistant to PTP induction by high light doses (Šileikyte et al., 2011) proving that the outer membrane can favor PTP opening through a still unidentified mechanism (Ricchelli et al., 2011).

Bcl-2 family proteins, which are generally believed to locate to the outer membrane, affect the PTP (Forte and Bernardi, 2006). According to several studies Bcl-2 increases resistance to the PT (Susin et al., 1996; Decaudin et al., 1997) while Bax and/or Bad activated by tBid favor PTP opening (Pastorino et al., 1998, 1999; Scorrano et al., 2002; Sinha Roy et al., 2009). Although CsA did not prevent cytochrome c release in one study where tBid was used, it is not known whether the cells expressed CyPD and were therefore sensitive to CsA inhibition (Garcia-Perez et al., 2012), an issue that may explain discrepancies with earlier studies (Scorrano et al., 2002; Tafani et al., 2002). Occurrence of a PT is instead opposed by outer membrane binding of hexokinase II (Pastorino et al., 2002, 2005; Chiara et al., 2008), which counteracts the inducing effects of Bax (Pastorino et al., 2002). It must be mentioned that Bcl-2 family proteins also interact with other targets. Indeed, tBid initiates a CsA-sensitive process of cristae remodeling that increases cytochrome c availability in the intermembrane space, which is consistent with a PT-dependent facilitation of cytochrome c release even when the outer membrane is intact (Scorrano et al., 2002, 2003); and Bax inhibits the mitochondrial inner membrane potassium channel Kv1.3 (Szabó et al., 2008), a proapoptotic effect that critically depends on Bax residue K128 and is abrogated by a K128E mutation, while an E158K mutation in antiapoptotic Bcl-xL (position 158 of Bcl-xL corresponds to position 128 of Bax) turns Bcl-xL into a proapoptotic protein (Szabó et al., 2011).

The detailed mechanisms through which the outer membrane affects formation of the PTP remain to be addressed. I would like to stress that the vast majority of complex V is located in long rows of oligomers deep inside mitochondrial cristae (Strauss et al., 2008; Davies et al., 2011, 2012) where direct physical interaction with the outer membrane is obviously not possible. Thus, either the PTP forms in the small population of dimers facing the intermembrane space, where direct contact with the outer membrane can occur; or the effect is exerted by controlling the diffusion of PTP-regulating metabolites and ions including Ca^2+^ itself (Madesh and Hajnoczky, 2001; Rapizzi et al., 2002) in a process that would be greatly favored by cristae remodeling (Scorrano et al., 2002).

### The PTP as a calcium release channel

We have recently reviewed the hypothesis (Bernardi and Petronilli, 1996; Ichas et al., 1997) that the PTP may open transiently (Hüser et al., 1998; Hüser and Blatter, 1999; Petronilli et al., 1999, 2001) to operate as a mitochondrial Ca^2+^ release channel under physiological conditions (Bernardi and von Stockum, 2012). The basis for this hypothesis goes back to 1992, when Altschuld et al. reported that CsA significantly increases net Ca^2+^ uptake and decreases Ca^2+^ efflux in isolated cardiomyocytes, as measured by radiolabeled ^45^Ca^2+^, through a demonstrably mitochondrial effect that has no impact on cell morphology or viability (Altschuld et al., 1992). Three recent publications based on *Ppif*^−/−^ cells and mice support this hypothesis (Elrod et al., 2010; Barsukova et al., 2011; Parone et al., 2013).

An age-related phenotype was discovered in the hearts of *Ppif*^−/−^ mice, which dysplayed decreased contractile reserve and increased shortening and relaxation times, with longer decay of cytosolic Ca^2+^ transients (Elrod et al., 2010). Direct measurement of total mitochondrial Ca^2+^ content of *Ppif*^−/−^ hearts showed a 2.6-fold increase, which was matched by larger Ca^2+^ transients in mitochondria of myocytes treated with CsA. Under continuous pacing PTP desensitization with CsA decreased the rise time in Ca^2+^ accumulation and prolonged the recovery time after pacing, findings that are entirely consistent with the PTP acting as a Ca^2+^ release channel to prevent Ca^2+^ overload (Elrod et al., 2010). Consistent with this idea, cytosolic [Ca^2+^] increases induced by either ATP or depolarizing concentrations of KCl gave comparable transient increases of mitochondrial [Ca^2+^] in adult cortical neurons from wild type and *Ppif*^−/−^ mice; while application of the two stimuli together resulted in much higher levels of mitochondrial [Ca^2+^] in the *Ppif*^−/−^ neurons, suggesting that the threshold for PTP activation had been reached in the wild type but not in the CyPD-null mitochondria *in situ*. These data indicate that the regulatory role of CyPD (and thus PTP opening) in Ca^2+^ homeostasis may be essential only for relatively large mitochondrial Ca^2+^ loads (Barsukova et al., 2011). Mice expressing amyotrophic lateral sclerosis-linked mutants of superoxide dismutase (SOD) 1 develop a motor neuron disease with many pathological hallmarks seen in patients (Gurney et al., 1994). Spinal cord mitochondria from these mice display decreased Ca^2+^ retention capacity long before onset of motor weakness and neuronal death (Damiano et al., 2006), and this was corrected by genetic elimination of the *Ppif*^−/−^ gene (Parone et al., 2013). Improved mitochondrial Ca^2+^ buffering was matched by improved mitochondrial ATP synthesis and reduced swelling, attenuation of glial activation, reduction of misfolded SOD1 aggregates in the spinal cord, and significant suppression of motor neuron death throughout disease, although survival was unfortunately not improved (Parone et al., 2013).

Transient PTP openings appear also to be at the basis of “superoxide flashes” observed with mitochondrially targeted, circularly permuted yellow fluorescent protein (mt-cpYFP) in a variety of cell types (Wang et al., 2008, 2013; Fang et al., 2011). There is an ongoing debate on whether the flashes are in fact partially or totally due to changes of matrix pH (Schwarzlander et al., 2011, 2012), although it is fair to say that the measured matrix alkalinization appears to be way too small to account for the fluorescence changes of mt-cpYFP (Wei-Lapierre et al., 2013).

The recent finding that Drosophila mitochondria possess a PTP-related but CsA-insensitive Ca^2+^ release channel selective for Ca^2+^ and H^+^ (von Stockum et al., 2011) provides a nice model system, and perhaps the missing link between the PTP of yeast and mammals (Azzolin et al., 2010). I am confident that it will be now possible to address the potential role of the PTP as a Ca^2+^ release channel through inhibition of the “pore function” of ATP synthase dimers through appropriate drugs and through genetic manipulation.

### Complex I

The PTP has the remarkable feature of being modulated by electron flow through complex I (Fontaine et al., 1998a), which may in part be linked to the fact that NADH oxidation favors PTP opening (Costantini et al., 1996). A specific role of complex I, however, is suggested by the striking discovery that in mammalian mitochondria rotenone can be an effective inhibitor of the pore (Chauvin et al., 2001; Li et al., 2012). Inhibition of the PT by rotenone is maximal in mitochondria from tissues that express low levels of CyPD, where CsA has little inhibitory effect; while inhibition by CsA is maximal in mitochondria from tissues with high levels of expression of CyPD, where rotenone does not affect the PTP. Finally, tissues with mitochondria expressing intermediate levels of CyPD are sensitive to both rotenone and CsA, with additive effects of the two inhibitors (Li et al., 2012). Intriguingly, the inhibitory effect of rotenone is potentiated by Pi, which is reminiscent of the Pi requirement for inhibition by CsA (Basso et al., 2008). The concentrations of rotenone required for PT inhibition matched quite precisely those required to inhibit respiration, and a similar PT-inhibiting effect was seen with metformin, which partially inhibits complex I as well (El Mir et al., 2000; Guigas et al., 2004). Since rotenone and metformin do not share common structural features, it appears likely that modulation of the PTP by inhibition of complex I is indirect, and possibly mediated by inhibition of complex I-derived reactive oxygen species (Batandier et al., 2004). An indirect effect is also suggested by the observations (1) that a Ca^2+^-dependent (albeit CsA-insensitive) PT can occur in mitochondria from yeast strains that do not possess an energy-conserving complex I (Jung et al., 1997; Yamada et al., 2009; Uribe-Carvajal et al., 2011); (2) that complex I purified by blue native electrophoresis did not form Ca^2+^-activated channels in lipid bilayers under conditions that instead promote MMC opening after incorporation of complex V (Giorgio et al., 2013); and (3) that complex I does not colocalize with ATP synthase oligomers (Davies et al., 2011).

### Quinones

The regulatory effects of quinones on the PTP has been discovered in our laboratory in 1998 (Fontaine et al., 1998a,b) and thoroughly characterized by Fontaine and Coworkers (Walter et al., 2000, 2002; Devun et al., 2010). Ub0 (i.e., ubiquinone without a side chain) is the most potent inhibitor of the PTP; the dose-dependence displays a bell-shaped curve, however, a feature that is also typical of other inhibitory quinones like decylubiquinone (Fontaine et al., 1998a,b; Fontaine and Bernardi, 1999; Walter et al., 2000). PTP-inducing quinones include idebenone (Walter et al., 2000) while idebenol (the reduced form) is no longer effective, suggesting that the effect on the PTP is related to oxidation-reduction events (Giorgio et al., 2012). The structure–function relationship of quinones for PTP inhibition/activation has not been solved, and further complexity arises from the fact that the same quinone can have opposite effects depending on the cell type (Devun et al., 2010). It appears possible that, like substituted maleimides, Ub0 affects PTP formation in part at least through its ability to form adducts with SH groups, which is not shared by short-chain quinones. ROS-forming ability of the latter does not always correlate with their PTP-inducing features (Devun et al., 2010) and the general mechanism of action of quinones on the PT remains to be solved.

## Conclusions and perspectives

The findings that (1) dimers of the ATP synthase treated with Ca^2+^ generate currents indistinguishable from the MMC–PTP; (2) channel openings are favored by Bz-423 and thiol oxidants; (3) channel openings are inhibited by adenine nucleotides and Mg^2+^; and (4) monomers lack any channel activity, all represent strong indications that the PTP forms from a specific, Ca^2+^-dependent conformation of the dimers. The F_O_F_1_ ATP synthase readily accommodates key pathophysiological effectors of the PT since Ca^2+^, Mg^2+^, adenine nucleotides, and Pi bind the catalytic core at F_1_; and the membrane potential and matrix pH, which are key PTP modulators (Bernardi et al., 2006), are also key regulators of the ATP synthase. On the other hand, several regulators of the PTP (specifically, rotenone and quinones) are not easily accounted for by the dimer hypothesis, and their mechanism of action will need further studies. However, I believe that our findings provide a novel and stimulating working hypothesis that should help address outstanding issues including species-specific features of the PTP of rats (Ricchelli et al., 2005; Zulian et al., 2007), yeast (Jung et al., 1997; Manon et al., 1998; Yamada et al., 2009; Uribe-Carvajal et al., 2011), and *Drosophila melanogaster* (von Stockum et al., 2011); and the mystery of *Artemia franciscana*, an anoxia and salt-tolerant brine shrimp whose mitochondria are refractory to PTP opening (Menze et al., 2005, 2010). Together our results suggest a dual function for complex V, ATP synthesis and PTP formation. The key enzyme of life appears therefore to be also the molecular switch that signals the presence of fully depolarized, dysfunctional mitochondria to promote cell death (Rasola and Bernardi, 2011) and/or mitophagy (Youle and Narendra, 2011).

### Conflict of interest statement

The author declares that the research was conducted in the absence of any commercial or financial relationships that could be construed as a potential conflict of interest.

## References

[B1] Adams V. Bosch W. Schlegel J. Wallimann T. Brdiczka D. (1989). Further characterization of contact sites from mitochondria of different tissues: topology of peripheral kinases. Biochim. Biophys. Acta 981, 213–225 2543459 10.1016/0005-2736(89)90031-x

[B2] Al Nasser I. Crompton M. (1986). The reversible Ca^2+^-induced permeabilization of rat liver mitochondria. Biochem. J. 239, 19–29 3099778 10.1042/bj2390019 PMC1147234

[B3] Altschuld R. A. Hohl C. M. Castillo L. C. Garleb A. A. Starling R. C. Brierley G. P. (1992). Cyclosporin inhibits mitochondrial calcium efflux in isolated adult rat ventricular cardiomyocytes. Am. J. Physiol. 262, H1699–H1704 1377876 10.1152/ajpheart.1992.262.6.H1699

[B4] Anholt R. R. De Souza E. B. Oster-Granite M. L. Snyder S. H. (1985). Peripheral-type benzodiazepine receptors: autoradiographic localization in whole-body sections of neonatal rats. J. Pharmacol. Exp. Ther. 233, 517–526 2987488

[B5] Anholt R. R. Pedersen P. L. De Souza E. B. Snyder S. H. (1986). The peripheral-type benzodiazepine receptor. Localization to the mitochondrial outer membrane. J. Biol. Chem. 261, 576–583 3001071

[B6] Azzi A. Azzone G. F. (1965). Swelling and shrinkage phenomena in liver mitochondria. I. Large amplitude swelling induced by inorganic phosphate and by ATP. Biochim. Biophys. Acta 105, 253–264 5849818 10.1016/s0926-6593(65)80150-3

[B7] Azzolin L. von Stockum S. Basso E. Petronilli V. Forte M. A. Bernardi P. (2010). The mitochondrial permeability transition from yeast to mammals. FEBS Lett. 584, 2504–2509 10.1016/j.febslet.2010.04.023 20398660 PMC2878904

[B8] Azzone G. F. Azzi A. (1965). Volume changes in liver mitochondria. Proc. Natl. Acad. Sci. U.S.A. 53, 1084–1089 5222552 10.1073/pnas.53.5.1084 PMC301376

[B9] Baines C. P. Kaiser R. A. Purcell N. H. Blair N. S. Osinska H. Hambleton M. A. (2005). Loss of cyclophilin D reveals a critical role for mitochondrial permeability transition in cell death. Nature 434, 658–662 10.1038/nature03434 15800627

[B10] Baines C. P. Kaiser R. A. Sheiko T. Craigen W. J. Molkentin J. D. (2007). Voltage-dependent anion channels are dispensable for mitochondrial-dependent cell death. Nat. Cell Biol. 9, 550–555 10.1038/ncb1575 17417626 PMC2680246

[B11] Baker L. A. Watt I. N. Runswick M. J. Walker J. E. Rubinstein J. L. (2012). Arrangement of subunits in intact mammalian mitochondrial ATP synthase determined by cryo-EM. Proc. Natl. Acad. Sci. U.S.A. 109, 11675–11680 10.1073/pnas.1204935109 22753497 PMC3406826

[B12] Barsukova A. Komarov A. Hajnoczky G. Bernardi P. Bourdette D. Forte M. (2011). Activation of the mitochondrial permeability transition pore modulates Ca^2+^ responses to physiological stimuli in adult neurons. Eur. J. Neurosci. 33, 831–842 10.1111/j.1460-9568.2010.07576.x 21255127 PMC3183752

[B13] Basso E. Fante L. Fowlkes J. Petronilli V. Forte M. A. Bernardi P. (2005). Properties of the permeability transition pore in mitochondria devoid of Cyclophilin, D. J. Biol. Chem. 280, 18558–18561 10.1074/jbc.C500089200 15792954

[B14] Basso E. Petronilli V. Forte M. A. Bernardi P. (2008). Phosphate is essential for inhibition of the mitochondrial permeability transition pore by cyclosporin A and by cyclophilin D ablation. J. Biol. Chem. 283, 26307–26311 10.1074/jbc.C800132200 18684715 PMC2546556

[B15] Batandier C. Leverve X. Fontaine E. (2004). Opening of the mitochondrial permeability transition pore induces reactive oxygen species production at the level of the respiratory chain complex, I. J. Biol. Chem. 279, 17197–17204 10.1074/jbc.M310329200 14963044

[B16] Batarseh A. Papadopoulos V. (2010). Regulation of translocator protein 18kDa (TSPO) expression in health and disease states. Mol. Cell. Endocrinol. 327, 1–12 10.1016/j.mce.2010.06.013 20600583 PMC2922062

[B17] Beatrice M. C. Palmer J. W. Pfeiffer D. R. (1980). The relationship between mitochondrial membrane permeability, membrane potential, and the retention of Ca^2+^ by mitochondria. J. Biol. Chem. 255, 8663–8671 7410387

[B18] Bernardi P. (1992). Modulation of the mitochondrial cyclosporin A-sensitive permeability transition pore by the proton electrochemical gradient. Evidence that the pore can be opened by membrane depolarization. J. Biol. Chem. 267, 8834–8839 1374381

[B19] Bernardi P. (1999). Mitochondrial transport of cations: channels, exchangers and permeability transition. Physiol. Rev. 79, 1127–1155 10508231 10.1152/physrev.1999.79.4.1127

[B20] Bernardi P. Krauskopf A. Basso E. Petronilli V. Blachly-Dyson E. Di Lisa F. (2006). The mitochondrial permeability transition from *in vitro* artifact to disease target. FEBS J. 273, 2077–2099 10.1111/j.1742-4658.2006.05213.x 16649987

[B21] Bernardi P. Penzo D. Wojtczak L. (2002). Mitochondrial energy dissipation by fatty acids. Mechanisms and implications for cell death. Vitam. Horm. 65, 97–126 12481544 10.1016/s0083-6729(02)65061-7

[B22] Bernardi P. Petronilli V. (1996). The permeability transition pore as a mitochondrial calcium release channel: a critical appraisal. J. Bioenerg. Biomembr. 28, 131–138 9132411 10.1007/BF02110643

[B23] Bernardi P. Pietrobon D. (1982). On the nature of Pi-induced, Mg^2+^-prevented Ca^2+^ release in rat liver mitochondria. FEBS Lett. 139, 9–12 10.1016/0014-5793(82)80475-4 7075769

[B24] Bernardi P. Vassanelli S. Veronese P. Colonna R. Szabo I. Zoratti M. (1992). Modulation of the mitochondrial permeability transition pore. Effect of protons and divalent cations. J. Biol. Chem. 267, 2934–2939 1737749

[B25] Bernardi P. von Stockum S. (2012). The permeability transition pore as a Ca^2+^ release channel: new answers to an old question. Cell Calcium 52, 22–27 10.1016/j.ceca.2012.03.004 22513364 PMC3396848

[B26] Beutner G. Rück A. Riede B. Welte W. Brdiczka D. (1996). Complexes between kinases, mitochondrial porin and adenylate translocator in rat brain resemble the permeability transition pore. FEBS Lett. 396, 189–195 10.1016/0014-5793(96)01092-7 8914985

[B27] Blatt N. B. Bednarski J. J. Warner R. E. Leonetti F. Johnson K. M. Boitano A. (2002). Benzodiazepine-induced superoxide signals B cell apoptosis: mechanistic insight and potential therapeutic utility. J. Clin. Invest. 110, 1123–1132 10.1172/JCI16029 12393848 PMC150800

[B28] Borel J. F. Feurer C. Magnee C. Stahelin H. (1977). Effects of the new anti-lymphocytic peptide cyclosporin A in animals. Immunology 32, 1017–1025 328380 PMC1445439

[B29] Brenner-Holzach O. Raaflaub J. (1954). Die korrelation zwischen der schwellung isolierter mitochondrien und dem abbau intramitochondrialen adenosinnucleotide (ATP, ADP, AMP, CoA). Helv. Physiol. Pharmacol. Acta 12, 242–252 13221178

[B30] Broekemeier K. M. Carpenter Deyo L. Reed D. J. Pfeiffer D. R. (1992). Cyclosporin A protects hepatocytes subjected to high Ca^2+^ and oxidative stress. FEBS Lett. 304, 192–194 1618322 10.1016/0014-5793(92)80616-o

[B31] Broekemeier K. M. Dempsey M. E. Pfeiffer D. R. (1989). Cyclosporin A is a potent inhibitor of the inner membrane permeability transition in liver mitochondria. J. Biol. Chem. 264, 7826–7830 2470734

[B32] Bucheler K. Adams V. Brdiczka D. (1991). Localization of the ATP/ADP translocator in the inner membrane and regulation of contact sites between mitochondrial envelope membranes by ADP. A study on freeze-fractured isolated liver mitochondria. Biochim. Biophys. Acta 1056, 233–242 1825787 10.1016/s0005-2728(05)80054-4

[B33] Campanella M. Casswell E. Chong S. Farah Z. Wieckowski M. R. Abramov A. Y. (2008). Regulation of mitochondrial structure and function by the F1Fo-ATPase inhibitor protein, IF1. Cell Metab. 8, 13–25 10.1016/j.cmet.2008.06.001 18590689

[B34] Chappell J. B. Crofts A. R. (1965). Calcium ion accumulation and volume changes of isolated liver mitochondria. Calcium ion-induced swelling. Biochem. J. 95, 378–386 14340088 10.1042/bj0950378 PMC1214334

[B35] Chauvin C. De Oliveira F. Ronot X. Mousseau M. Leverve X. Fontaine E. (2001). Rotenone inhibits the mitochondrial permeability transition-induced cell death in U937 and KB cells. J. Biol. Chem. 276, 41394–41398 10.1074/jbc.M106417200 11527970

[B36] Chiara F. Castellaro D. Marin O. Petronilli V. Brusilow W. S. Juhaszova M. (2008). Hexokinase II detachment from mitochondria triggers apoptosis through the permeability transition pore independent of voltage-dependent anion channels. PLoS ONE 3:e1852 10.1371/journal.pone.0001852 18350175 PMC2267038

[B37] Cleary J. Johnson K. M. Opipari A. W. Jr. Glick G. D. (2007). Inhibition of the mitochondrial F1F0-ATPase by ligands of the peripheral benzodiazepine receptor. Bioorg. Med. Chem. Lett. 17, 1667–1670 10.1016/j.bmcl.2006.12.102 17251020

[B38] Clipstone N. A. Crabtree G. R. (1992). Identification of calcineurin as a key signalling enzyme in T-lymphocyte activation. Nature 357, 695–697 10.1038/357695a0 1377362

[B39] Coelho J. L. Vercesi A. E. (1980). Retention of Ca^2+^ by rat liver and rat heart mitochondria: effect of phosphate, Mg^2+^, and NAD(P) redox state. Arch. Biochem. Biophys. 204, 141–147 10.1016/0003-9861(80)90016-8 7425633

[B40] Connern C. P. Halestrap A. P. (1992). Purification and N-terminal sequencing of peptidyl-prolyl cis-trans-isomerase from rat liver mitochondrial matrix reveals the existence of a distinct mitochondrial cyclophilin. Biochem. J. 284, 381–385 1599421 10.1042/bj2840381 PMC1132649

[B41] Connern C. P. Halestrap A. P. (1994). Recruitment of mitochondrial cyclophilin to the mitochondrial inner membrane under conditions of oxidative stress that enhance the opening of a calcium-sensitive non-specific channel. Biochem. J. 302, 321–324 7522435 10.1042/bj3020321 PMC1137230

[B42] Costantini P. Chernyak B. V. Petronilli V. Bernardi P. (1996). Modulation of the mitochondrial permeability transition pore by pyridine nucleotides and dithiol oxidation at two separate sites. J. Biol. Chem. 271, 6746–6751 10.1074/jbc.271.12.6746 8636095

[B43] Costantini P. Colonna R. Bernardi P. (1998). Induction of the mitochondrial permeability transition by N-ethylmaleimide depends on secondary oxidation of critical thiol groups. Potentiation by copper-ortho-phenanthroline without dimerization of the adenine nucleotide translocase. Biochim. Biophys. Acta 1365, 385–392 10.1016/S0005-2728(98)00090-5 9711294

[B44] Crofts A. R. Chappell J. B. (1965). Calcium ion accumulation and volume changes of isolated rat liver mitochondria. Reversal of calcium ion-induced swelling. Biochem. J. 95, 387–392 14340089 10.1042/bj0950387 PMC1214335

[B45] Crompton M. Costi A. (1988). Kinetic evidence for a heart mitochondrial pore activated by Ca^2+^, inorganic phosphate and oxidative stress. A potential mechanism for mitochondrial dysfunction during cellular Ca^2+^ overload. Eur. J. Biochem. 178, 489–501 10.1111/j.1432-1033.1988.tb14475.x 2850179

[B46] Crompton M. Ellinger H. Costi A. (1988). Inhibition by cyclosporin A of a Ca^2+^-dependent pore in heart mitochondria activated by inorganic phosphate and oxidative stress. Biochem. J. 255, 357–360 3196322 PMC1135230

[B47] Csukly K. Ascah A. Matas J. Gardiner P. F. Fontaine E. Burelle Y. (2006). Muscle denervation promotes opening of the permeability transition pore and increases the expression of cyclophilin D. J. Physiol. 574, 319–327 10.1113/jphysiol.2006.109702 16675492 PMC1817793

[B48] Damiano M. Starkov A. A. Petri S. Kipiani K. Kiaei M. Mattiazzi M. (2006). Neural mitochondrial Ca^2+^ capacity impairment precedes the onset of motor symptoms in G93A Cu/Zn-superoxide dismutase mutant mice. J. Neurochem. 96, 1349–1361 10.1111/j.1471-4159.2006.03619.x 16478527

[B49] Davidson A. M. Halestrap A. P. (1990). Partial inhibition by cyclosporin A of the swelling of liver mitochondria *in vivo* and *in vitro* induced by sub-micromolar [Ca^2+^], but not by butyrate. Evidence for two distinct swelling mechanisms. Biochem. J. 268, 147–152 2344354 10.1042/bj2680147 PMC1131404

[B50] Davies K. M. Anselmi C. Wittig I. Faraldo-Gomez J. D. Kühlbrandt W. (2012). Structure of the yeast F1Fo-ATP synthase dimer and its role in shaping the mitochondrial cristae. Proc. Natl. Acad. Sci. U.S.A. 109, 13602–13607 10.1073/pnas.1204593109 22864911 PMC3427116

[B51] Davies K. M. Strauss M. Daum B. Kief J. H. Osiewacz H. D. Rycovska A. (2011). Macromolecular organization of ATP synthase and complex I in whole mitochondria. Proc. Natl. Acad. Sci. U.S.A. 108, 14121–14126 10.1073/pnas.1103621108 21836051 PMC3161574

[B51a] Decaudin D. Geley S. Hirsch T. Castedo M. Marchetti P. Macho A. (1997). Bcl-2 and Bcl-XL antagonize the mitochondrial dysfunction preceding nuclear apoptosis induced by chemotherapeutic agents. Cancer Res. 57, 62–67 8988042

[B52] De Marchi U. Basso E. Szabó I. Zoratti M. (2006). Electrophysiological characterization of the Cyclophilin D-deleted mitochondrial permeability transition pore. Mol. Membr. Biol. 23, 521–530 10.1080/09687860600907644 17127624

[B53] Devun F. Walter L. Belliere J. Cottet-Rousselle C. Leverve X. Fontaine E. (2010). Ubiquinone analogs: a mitochondrial permeability transition pore-dependent pathway to selective cell death. PLoS ONE 5:e11792 10.1371/journal.pone.0011792 20668684 PMC2909912

[B54] Du C. Fang M. Li Y. Li L. Wang X. (2000). Smac, a mitochondrial protein that promotes cytochrome c-dependent caspase activation by eliminating IAP inhibition. Cell 102, 33–42 10.1016/S0092-8674(00)00008-8 10929711

[B55] Duchen M. R. McGuinness O. Brown L. A. Crompton M. (1993). On the involvement of a cyclosporin A sensitive mitochondrial pore in myocardial reperfusion injury. Cardiovasc. Res. 27, 1790–1794 10.1093/cvr/27.10.1790 8275525

[B56] Ekert P. G. Silke J. Connolly L. M. Reid G. E. Moritz R. L. Vaux D. L. (2000). Identification of DIABLO, a mammalian protein that promotes apoptosis by binding to and antagonizing IAP proteins. Cell 102, 43–53 10.1016/S0092-8674(00)00009-X 10929712

[B57] Eliseev R. A. Malecki J. Lester T. Zhang Y. Humphrey J. Gunter T. E. (2009). Cyclophilin D interacts with Bcl2 and exerts an anti-apoptotic effect. J. Biol. Chem. 284, 9692–9699 10.1074/jbc.M808750200 19228691 PMC2665090

[B58] El Mir M. Y. Nogueira V. Fontaine E. Averet N. Rigoulet M. Leverve X. (2000). Dimethylbiguanide inhibits cell respiration via an indirect effect targeted on the respiratory chain complex I. J. Biol. Chem. 275, 223–228 10.1074/jbc.275.1.223 10617608

[B59] Elrod J. W. Wong R. Mishra S. Vagnozzi R. J. Sakthievel B. Goonasekera S. A. (2010). Cyclophilin D controls mitochondrial pore-dependent Ca^2+^ exchange, metabolic flexibility, and propensity for heart failure in mice. J. Clin. Invest. 120, 3680–3687 10.1172/JCI43171 20890047 PMC2947235

[B60] Fang H. Chen M. Ding Y. Shang W. Xu J. Zhang X. (2011). Imaging superoxide flash and metabolism-coupled mitochondrial permeability transition in living animals. Cell Res. 21, 1295–1304 10.1038/cr.2011.81 21556035 PMC3193463

[B61] Fischer G. Wittmann-Liebold B. Lang K. Kiefhaber T. Schmid F. X. (1989). Cyclophilin and peptidyl-prolyl cis-trans isomerase are probably identical proteins. Nature 337, 476–478 10.1038/337476a0 2492638

[B62] Fontaine E. Bernardi P. (1999). Progress on the mitochondrial permeability transition pore: regulation by complex I and ubiquinone analogs. J. Bioenerg. Biomembr. 31, 335–345 10665524 10.1023/a:1005475802350

[B63] Fontaine E. Eriksson O. Ichas F. Bernardi P. (1998a). Regulation of the permeability transition pore in skeletal muscle mitochondria. Modulation by electron flow through the respiratory chain complex I. J. Biol. Chem. 273, 12662–12668 10.1074/jbc.273.20.12662 9575229

[B64] Fontaine E. Ichas F. Bernardi P. (1998b). A ubiquinone-binding site regulates the mitochondrial permeability transition pore. J. Biol. Chem. 273, 25734–25740 10.1074/jbc.273.40.25734 9748242

[B65] Forte M. Bernardi P. (2006). The permeability transition and BCL-2 family proteins in apoptosis: co-conspirators or independent agents? Cell Death Differ. 13, 1287–1290 10.1038/sj.cdd.4401957 16691209

[B66] Fournier N. Ducet G. Crevat A. (1987). Action of cyclosporine on mitochondrial calcium fluxes. J. Bioenerg. Biomembr. 19, 297–303 3114244 10.1007/BF00762419

[B67] Garcia-Perez C. Roy S. S. Naghdi S. Lin X. Davies E. Hajnoczky G. (2012). Bid-induced mitochondrial membrane permeabilization waves propagated by local reactive oxygen species (ROS) signaling. Proc. Natl. Acad. Sci. U.S.A. 109, 4497–4502 10.1073/pnas.1118244109 22393005 PMC3311374

[B68] Giorgio V. Bisetto E. Soriano M. E. Dabbeni-Sala F. Basso E. Petronilli V. (2009). Cyclophilin D modulates mitochondrial F_O_F_1_-ATP synthase by interacting with the lateral stalk of the complex. J. Biol. Chem. 284, 33982–33988 10.1074/jbc.M109.020115 19801635 PMC2797168

[B69] Giorgio V. Petronilli V. Ghelli A. Carelli V. Rugolo M. Lenaz G. (2012). The effects of idebenone on mitochondrial bioenergetics. Biochim. Biophys. Acta 1817, 363–369 10.1016/j.bbabio.2011.10.012 22086148 PMC3265671

[B70] Giorgio V. Soriano M. E. Basso E. Bisetto E. Lippe G. Forte M. A. (2010). Cyclophilin D in mitochondrial pathophysiology. Biochim. Biophys. Acta 1797, 1113–1118 10.1016/j.bbabio.2009.12.006 20026006 PMC2888675

[B71] Giorgio V. von Stockum S. Antoniel M. Fabbro A. Fogolari F. Forte M. (2013). Dimers of mitochondrial ATP synthase form the permeability transition pore. Proc. Natl. Acad. Sci. U.S.A. 110, 5887–5892 10.1073/pnas.1217823110 23530243 PMC3625323

[B72] Griffiths E. J. Halestrap A. P. (1995). Mitochondrial non-specific pores remain closed during cardiac ischaemia, but open upon reperfusion. Biochem. J. 307, 93–98 7717999 10.1042/bj3070093 PMC1136749

[B73] Guigas B. Detaille D. Chauvin C. Batandier C. De Oliveira F. Fontaine E. (2004). Metformin inhibits mitochondrial permeability transition and cell death: a pharmacological *in vitro* study. Biochem. J. 382, 877–884 10.1042/BJ20040885 15175014 PMC1133963

[B74] Gunter T. E. Pfeiffer D. R. (1990). Mechanisms by which mitochondria transport calcium. Am. J. Physiol. 258, C755–C786 2185657 10.1152/ajpcell.1990.258.5.C755

[B75] Gurney M. E. Pu H. Chiu A. Y. Dal Canto M. C. Polchow C. Y. Alexander D. D. (1994). Motor neuron degeneration in mice that express a human Cu, Zn superoxide dismutase mutation. Science 264, 1772–1775 10.1126/science.8209258 8209258

[B76] Halestrap A. P. (2004). Mitochondrial permeability: dual role for the ADP/ATP translocator? Nature 430, 1 p following 983. 15332302 10.1038/nature02816

[B77] Haworth R. A. Hunter D. R. (1979). The Ca^2+^-induced membrane transition of rat liver mitochondria. II. Nature of the Ca^2+^ trigger site. Arch. Biochem. Biophys. 195, 460–467 38751 10.1016/0003-9861(79)90372-2

[B78] He L. Lemasters J. J. (2002). Regulated and unregulated mitochondrial permeability transition pores: a new paradigm of pore structure and function? FEBS Lett. 512, 1–7 11852041 10.1016/s0014-5793(01)03314-2

[B79] Hegde R. Srinivasula S. M. Zhang Z. Wassell R. Mukattash R. Cilenti L. (2001). Identification of Omi/HtrA2 as a mitochondrial apoptotic serine protease that disrupts inhibitor of apoptosis protein-caspase interaction. J. Biol. Chem. 277, 432–438 10.1074/jbc.M109721200 11606597

[B80] Herick K. Krämer R. Lühring H. (1997). Patch clamp investigation into the phosphate carrier from *Saccharomyces cerevisiae* mitochondria. Biochim. Biophys. Acta 1321, 207–220 10.1016/S0005-2728(97)00050-9 9393638

[B81] Hunter D. R. Haworth R. A. (1979a). The Ca^2+^-induced membrane transition in mitochondria. I. The protective mechanisms. Arch. Biochem. Biophys. 195, 453–459 383019 10.1016/0003-9861(79)90371-0

[B82] Hunter D. R. Haworth R. A. (1979b). The Ca^2+^-induced membrane transition in mitochondria. III. Transitional Ca^2+^ release. Arch. Biochem. Biophys. 195, 468–477 112926 10.1016/0003-9861(79)90373-4

[B83] Hunter D. R. Haworth R. A. Southard J. H. (1976). Relationship between configuration, function, and permeability in calcium-treated mitochondria. J. Biol. Chem. 251, 5069–5077 134035

[B84] Hunter F. E. Jr. Ford L. (1955). Inactivation of oxidative and phosphorylative systems in mitochondria by preincubation with phosphate and other ions. J. Biol. Chem. 216, 357–369 13252035

[B85] Hüser J. Blatter L. A. (1999). Fluctuations in mitochondrial membrane potential caused by repetitive gating of the permeability transition pore. Biochem. J. 343, 311–317 10510294 PMC1220555

[B86] Hüser J. Rechenmacher C. E. Blatter L. A. (1998). Imaging the permeability pore transition in single mitochondria. Biophys. J. 74, 2129–2137 10.1016/S0006-3495(98)77920-2 9545072 PMC1299554

[B87] Ichas F. Jouaville L. S. Mazat J. P. (1997). Mitochondria are excitable organelles capable of generating and conveying electrical and calcium signals. Cell 89, 1145–1153 10.1016/S0092-8674(00)80301-3 9215636

[B88] Imberti R. Nieminen A. L. Herman B. Lemasters J. J. (1992). Synergism of cyclosporin A and phospholipase inhibitors in protection against lethal injury to rat hepatocytes from oxidant chemicals. Res. Commun. Chem. Pathol. Pharmacol. 78, 27–38 1462047

[B89] Johnson K. M. Chen X. Boitano A. Swenson L. Opipari A. W. Jr. Glick G. D. (2005). Identification and validation of the mitochondrial F1F0-ATPase as the molecular target of the immunomodulatory benzodiazepine Bz-423. Chem. Biol. 12, 485–496 10.1016/j.chembiol.2005.02.012 15850986

[B90] Jung D. W. Bradshaw P. C. Pfeiffer D. R. (1997). Properties of a cyclosporin-insensitive permeability transition pore in yeast mitochondria. J. Biol. Chem. 272, 21104–21112 10.1074/jbc.272.34.21104 9261114

[B91] Kang B. H. Plescia J. Dohi T. Rosa J. Doxsey S. J. Altieri D. C. (2007). Regulation of tumor cell mitochondrial homeostasis by an organelle-specific Hsp90 chaperone network. Cell 131, 257–270 10.1016/j.cell.2007.08.028 17956728

[B92] Karch J. Molkentin J. D. (2012). Is p53 the long-sought molecular trigger for cyclophilin D-regulated mitochondrial permeability transition pore formation and necrosis? Circ. Res. 111, 1258–1260 10.1161/CIRCRESAHA.112.280990 23104876

[B93] Kinnally K. W. Campo M. L. Tedeschi H. (1989). Mitochondrial channel activity studied by patch-clamping mitoplasts. J. Bioenerg. Biomembr. 21, 497–506 2478535 10.1007/BF00762521

[B94] Kinnally K. W. Zorov D. B. Antonenko Y. N. Snyder S. H. McEnery M. W. Tedeschi H. (1993). Mitochondrial benzodiazepine receptor linked to inner membrane ion channels by nanomolar actions of ligands. Proc. Natl. Acad. Sci. U.S.A. 90, 1374–1378 10.1073/pnas.90.4.1374 7679505 PMC45875

[B95] Kohr M. J. Aponte A. M. Sun J. Wang G. Murphy E. Gucek M. (2011). Characterization of potential S-nitrosylation sites in the myocardium. Am. J. Physiol. Heart Circ. Physiol. 300, H1327–H1335 10.1152/ajpheart.00997.2010 21278135 PMC3075037

[B96] Kokoszka J. E. Waymire K. G. Levy S. E. Sligh J. E. Cai J. Jones D. P. (2004). The ADP/ATP translocator is not essential for the mitochondrial permeability transition pore. Nature 427, 461–465 10.1038/nature02229 14749836 PMC3049806

[B97] Kottke M. Adam V. Riesinger I. Bremm G. Bosch W. Brdiczka D. (1988). Mitochondrial boundary membrane contact sites in brain: points of hexokinase and creatine kinase location, and control of Ca^2+^ transport. Biochim. Biophys. Acta 935, 87–102 10.1016/0005-2728(88)90111-9 2457393

[B98] Kowaltowski A. J. Castilho R. F. Vercesi A. E. (2001). Mitochondrial permeability transition and oxidative stress. FEBS Lett. 495, 12–15 11322939 10.1016/s0014-5793(01)02316-x

[B99] Krauskopf A. Eriksson O. Craigen W. J. Forte M. A. Bernardi P. (2006). Properties of the permeability transition in VDAC1(-/-) mitochondria. Biochim. Biophys. Acta 1757, 590–595 10.1016/j.bbabio.2006.02.007 16626625

[B100] Lehninger A. L. (1959). Reversal of various types of mitochondrial swelling by adenosine triphosphate. J. Biol. Chem. 234, 2465–2471 14415396

[B101] Lehninger A. L. Remmert L. F. (1959). An endogenous uncoupling and swelling agent in liver mitochondria and its enzymic function. J. Biol. Chem. 234, 2459–2464 14415393

[B102] Lê-Quôc K. Lê-Quôc D. (1982). Control of the mitochondrial inner membrane permeability by sulfhydryl groups. Arch. Biochem. Biophys. 216, 639–651 7114855 10.1016/0003-9861(82)90254-5

[B103] Lê-Quôc K. Lê-Quôc D. (1985). Crucial role of sulfhydryl groups in the mitochondrial inner membrane structure. J. Biol. Chem. 260, 7422–7428 3997877

[B104] Leung A. W. Halestrap A. P. (2008). Recent progress in elucidating the molecular mechanism of the mitochondrial permeability transition pore. Biochim. Biophys. Acta 1777, 946–952 10.1016/j.bbabio.2008.03.009 18407825

[B105] Leung A. W. Varanyuwatana P. Halestrap A. P. (2008). The mitochondrial phosphate carrier interacts with cyclophilin D and may play a key role in the permeability transition. J. Biol. Chem. 283, 26312–26323 10.1074/jbc.M805235200 18667415 PMC3258905

[B106] Li B. Chauvin C. De P. D. De O. F. Gharib A. Vial G. (2012). Inhibition of complex I regulates the mitochondrial permeability transition through a phosphate-sensitive inhibitory site masked by cyclophilin D. Biochim. Biophys. Acta 1817, 1628–1634 10.1016/j.bbabio.2012.05.011 22659400

[B107] Li L. Y. Luo X. Wang X. (2001). Endonuclease G is an apoptotic DNase when released from mitochondria. Nature 412, 95–99 10.1038/35083620 11452314

[B108] Liu J. Farmer J. D. J. Lane W. S. Friedman J. Weissman I. Schreiber S. L. (1991). Calcineurin is a common target of cyclophilin-cyclosporin A and FKBP-FK506 complexes. Cell 66, 807–815 10.1016/0092-8674(91)90124-H 1715244

[B109] Liu X. Kim C. N. Yang J. Jemmerson R. Wang X. (1996). Induction of apoptotic program in cell-free extracts: requirement for dATP and cytochrome c. Cell 86, 147–157 10.1016/S0092-8674(00)80085-9 8689682

[B110] Madesh M. Hajnoczky G. (2001). VDAC-dependent permeabilization of the outer mitochondrial membrane by superoxide induces rapid and massive cytochrome c release. J. Cell Biol. 155, 1003–1015 10.1083/jcb.200105057 11739410 PMC2150912

[B111] Manon S. Roucou X. Guerin M. Rigoulet M. Guerin B. (1998). Characterization of the yeast mitochondria unselective channel: a counterpart to the mammalian permeability transition pore? J. Bioenerg. Biomembr. 30, 419–429 9932645 10.1023/a:1020533928491

[B112] Marzo I. Brenner C. Zamzami N. Susin S. A. Beutner G. Brdiczka D. (1998). The permeability transition pore complex: a target for apoptosis regulation by caspases and bcl-2-related proteins. J. Exp. Med. 187, 1261–1271 10.1084/jem.187.8.1261 9547337 PMC2212234

[B113] Menze M. A. Fortner G. Nag S. Hand S. C. (2010). Mechanisms of apoptosis in Crustacea: what conditions induce versus suppress cell death? Apoptosis 15, 293–312 10.1007/s10495-009-0443-6 20043212 PMC4104421

[B114] Menze M. A. Hutchinson K. Laborde S. M. Hand S. C. (2005). Mitochondrial permeability transition in the crustacean *Artemia franciscana*: absence of a calcium-regulated pore in the face of profound calcium storage. Am. J. Physiol. 289, R68–R76 10.1152/ajpregu.00844.2004 15718386

[B115] Mironova G. D. Gateau-Roesch O. Levrat C. Gritsenko E. Pavlov E. Lazareva A. V. (2001). Palmitic and stearic acids bind Ca^2+^ with high affinity and form nonspecific channels in black-lipid membranes. Possible relation to Ca^2+^-activated mitochondrial pores. J. Bioenerg. Biomembr. 33, 319–331 11710807 10.1023/a:1010659323937

[B116] Mitchell P. (1961). Coupling of phosphorylation to electron and hydrogen transfer by a chemio-osmotic type of mechanism. Nature 191, 144–148 13771349 10.1038/191144a0

[B117] Mitchell P. (2011). Chemiosmotic coupling in oxidative and photosynthetic phosphorylation. 1966. Biochim. Biophys. Acta 1807, 1507–1538 10.1016/j.bbabio.2011.09.018 22082452

[B118] Moreno G. Poussin K. Ricchelli F. Salet C. (2001). The effects of singlet oxygen produced by photodynamic action on the mitochondrial permeability transition differ in accordance with the localization of the sensitizer. Arch. Biochem. Biophys. 386, 243–250 10.1006/abbi.2000.2200 11368348

[B119] Nakagawa T. Shimizu S. Watanabe T. Yamaguchi O. Otsu K. Yamagata H. (2005). Cyclophilin D-dependent mitochondrial permeability transition regulates some necrotic but not apoptotic cell death. Nature 434, 652–658 10.1038/nature03317 15800626

[B120] Nguyen T. T. Stevens M. V. Kohr M. Steenbergen C. Sack M. N. Murphy E. (2011). Cysteine 203 of cyclophilin D is critical for cyclophilin D activation of the mitochondrial permeability transition pore. J. Biol. Chem. 286, 40184–40192 10.1074/jbc.M111.243469 21930693 PMC3220546

[B121] Nicolli A. Basso E. Petronilli V. Wenger R. M. Bernardi P. (1996). Interactions of cyclophilin with the mitochondrial inner membrane and regulation of the permeability transition pore, a cyclosporin A-sensitive channel. J. Biol. Chem. 271, 2185–2192 10.1074/jbc.271.4.2185 8567677

[B122] Nicolli A. Petronilli V. Bernardi P. (1993). Modulation of the mitochondrial cyclosporin A-sensitive permeability transition pore by matrix pH. Evidence that the pore open-closed probability is regulated by reversible histidine protonation. Biochemistry 32, 4461–4465 7682848 10.1021/bi00067a039

[B123] Parone P. A. Da C. S. Han J. S. McAlonis-Downes M. Vetto A. P. Lee S. K. (2013). Enhancing mitochondrial calcium buffering capacity reduces aggregation of misfolded SOD1 and motor neuron cell death without extending survival in mouse models of inherited amyotrophic lateral sclerosis. J. Neurosci. 33, 4657–4671 10.1523/JNEUROSCI.1119-12.2013 23486940 PMC3711648

[B124] Pastorino J. G. Chen S. T. Tafani M. Snyder J. W. Farber J. L. (1998). The overexpression of Bax produces cell death upon induction of the mitochondrial permeability transition. J. Biol. Chem. 273, 7770–7775 10.1074/jbc.273.13.7770 9516487

[B125] Pastorino J. G. Hoek J. B. Shulga N. (2005). Activation of glycogen synthase kinase 3beta disrupts the binding of hexokinase II to mitochondria by phosphorylating voltage-dependent anion channel and potentiates chemotherapy-induced cytotoxicity. Cancer Res. 65, 10545–10554 10.1158/0008-5472.CAN-05-1925 16288047

[B126] Pastorino J. G. Shulga N. Hoek J. B. (2002). Mitochondrial binding of hexokinase II inhibits bax-induced cytochrome c release and apoptosis. J. Biol. Chem. 277, 7610–7618 10.1074/jbc.M109950200 11751859

[B127] Pastorino J. G. Simbula G. Gilfor E. Hoek J. B. Farber J. L. (1994). Protoporphyrin, IX, an endogenous ligand of the peripheral benzodiazepine receptor, potentiates induction of the mitochondrial permeability transition and the killing of cultured hepatocytes by rotenone. J. Biol. Chem. 269, 31041–31046 7983042

[B128] Pastorino J. G. Snyder J. W. Serroni A. Hoek J. B. Farber J. L. (1993). Cyclosporin and carnitine prevent the anoxic death of cultured hepatocytes by inhibiting the mitochondrial permeability transition. J. Biol. Chem. 268, 13791–13798 8314748

[B129] Pastorino J. G. Tafani M. Rothman R. J. Marcineviciute A. Hoek J. B. Farber J. L. (1999). Functional consequences of the sustained or transient activation by Bax of the mitochondrial permeability transition pore. J. Biol. Chem. 274, 31734–31739 10.1074/jbc.274.44.31734 10531385

[B130] Pavlov E. Zakharian E. Bladen C. Diao C. T. Grimbly C. Reusch R. N. (2005). A large, voltage-dependent channel, isolated from mitochondria by water-free chloroform extraction. Biophys. J. 88, 2614–2625 10.1529/biophysj.104.057281 15695627 PMC1305358

[B131] Petronilli V. Miotto G. Canton M. Brini M. Colonna R. Bernardi P. (1999). Transient and long-lasting openings of the mitochondrial permeability transition pore can be monitored directly in intact cells by changes in mitochondrial calcein fluorescence. Biophys. J. 76, 725–734 10.1016/S0006-3495(99)77239-5 9929477 PMC1300077

[B132] Petronilli V. Nicolli A. Costantini P. Colonna R. Bernardi P. (1994). Regulation of the permeability transition pore, a voltage-dependent mitochondrial channel inhibited by cyclosporin A. Biochim. Biophys. Acta 1187, 255–259 7521212 10.1016/0005-2728(94)90122-8

[B133] Petronilli V. Penzo D. Scorrano L. Bernardi P. Di Lisa F. (2001). The mitochondrial permeability transition, release of cytochrome c and cell death. Correlation with the duration of pore openings *in situ*. J. Biol. Chem. 276, 12030–12034 10.1074/jbc.M010604200 11134038

[B134] Petronilli V. Šileikyte J. Zulian A. Dabbeni-Sala F. Jori G. Gobbo S. (2009). Switch from inhibition to activation of the mitochondrial permeability transition during hematoporphyrin-mediated photooxidative stress. Unmasking pore-regulating external thiols. Biochim. Biophys. Acta 1787, 897–904 10.1016/j.bbabio.2009.03.014 19344690

[B135] Petronilli V. Szabó I. Zoratti M. (1989). The inner mitochondrial membrane contains ion-conducting channels similar to those found in bacteria. FEBS Lett. 259, 137–143 10.1016/0014-5793(89)81513-3 2480918

[B136] Pfeiffer D. R. Kuo T. H. Tchen T. T. (1976). Some effects of Ca^2+^, Mg^2+^, and Mn^2+^ on the ultrastructure, light-scattering properties, and malic enzyme activity of adrenal cortex mitochondria. Arch. Biochem. Biophys. 176, 556–563 984848 10.1016/0003-9861(76)90199-5

[B137] Pfeiffer D. R. Tchen T. T. (1973). The role of Ca^2+^ in control of malic enzyme activity in bovine adrenal cortex mitochondria. Biochem. Biophys. Res. Commun. 50, 807–813 10.1016/0006-291X(73)91316-8 4144008

[B138] Pfeiffer D. R. Tchen T. T. (1975). The activation of adrenal cortex mitochondrial malic enzyme by Ca^2+^ and Mg^2+^. Biochemistry 14, 89–96 1167337 10.1021/bi00672a015

[B139] Raaflaub J. (1953a). Die schwellung isolierter leberzell mitochondrien und ihre physikalisch beeinfluβarkeit. Helv. Physiol. Pharmacol. Acta 11, 142–156 13095885

[B140] Raaflaub J. (1953b). Über den wirkungsmechanismus von adenosintriphosphat (ATP) als cofaktor isolierter mitochondrien. Helv. Physiol. Pharmacol. Acta 11, 157–165 13095886

[B141] Rapizzi E. Pinton P. Szabadkai G. Wieckowski M. R. Vandecasteele G. Baird G. (2002). Recombinant expression of the voltage-dependent anion channel enhances the transfer of Ca^2+^ microdomains to mitochondria. J. Cell Biol. 159, 613–624 10.1083/jcb.200205091 12438411 PMC2173108

[B142] Rasola A. Bernardi P. (2007). The mitochondrial permeability transition pore and its involvement in cell death and in disease pathogenesis. Apoptosis 12, 815–833 10.1007/s10495-007-0723-y 17294078

[B143] Rasola A. Bernardi P. (2011). Mitochondrial permeability transition in Ca^2+^-dependent apoptosis and necrosis. Cell Calcium 50, 222–233 10.1016/j.ceca.2011.04.007 21601280

[B144] Rasola A. Sciacovelli M. Chiara F. Pantic B. Brusilow W. S. Bernardi P. (2010). Activation of mitochondrial ERK protects cancer cells from death through inhibition of the permeability transition. Proc. Natl. Acad. Sci. U.S.A. 107, 726–731 10.1073/pnas.0912742107 20080742 PMC2818893

[B145] Rees D. M. Leslie A. G. Walker J. E. (2009). The structure of the membrane extrinsic region of bovine ATP synthase. Proc. Natl. Acad. Sci. U.S.A. 106, 21597–21601 10.1073/pnas.0910365106 19995987 PMC2789756

[B146] Ricchelli F. Dabbeni-Sala F. Petronilli V. Bernardi P. Hopkins B. Bova S. (2005). Species-specific modulation of the mitochondrial permeability transition by norbormide. Biochim. Biophys. Acta 1708, 178–186 10.1016/j.bbabio.2005.03.002 15953474

[B147] Ricchelli F. Šileikyte J. Bernardi P. (2011). Shedding light on the mitochondrial permeability transition. Biochim. Biophys. Acta 1807, 482–490 10.1016/j.bbabio.2011.02.012 21377443

[B148] Riley W. W. Jr. Pfeiffer D. R. (1985). Relationships between Ca^2+^ release, Ca^2+^ cycling, and Ca^2+^- mediated permeability changes in mitochondria. J. Biol. Chem. 260, 12416–12425 2413023

[B149] Salet C. Moreno G. Ricchelli F. Bernardi P. (1997). Singlet oxygen produced by photodynamic action causes inactivation of the mitochondrial permeability transition pore. J. Biol. Chem. 272, 21938–21943 10.1074/jbc.272.35.21938 9268328

[B150] Schinzel A. C. Takeuchi O. Huang Z. Fisher J. K. Zhou Z. Rubens J. (2005). Cyclophilin D is a component of mitochondrial permeability transition and mediates neuronal cell death after focal cerebral ischemia. Proc. Natl. Acad. Sci. U.S.A. 102, 12005–12010 10.1073/pnas.0505294102 16103352 PMC1189333

[B151] Schwarzlander M. Logan D. C. Fricker M. D. Sweetlove L. J. (2011). The circularly permuted yellow fluorescent protein cpYFP that has been used as a superoxide probe is highly responsive to pH but not superoxide in mitochondria: implications for the existence of superoxide ‘flashes’. Biochem. J. 437, 381–387 10.1042/BJ20110883 21631430

[B152] Schwarzlander M. Murphy M. P. Duchen M. R. Logan D. C. Fricker M. D. Halestrap A. P. (2012). Mitochondrial ‘flashes’: a radical concept repHined. Trends Cell Biol. 22, 503–508 10.1016/j.tcb.2012.07.007 22917552

[B153] Scorrano L. Ashiya M. Buttle K. Weiler S. Oakes S. A. Mannella C. A. (2002). A distinct pathway remodels mitochondrial cristae and mobilizes cytochrome c during apoptosis. Dev. Cell 2, 55–67 10.1016/S1534-5807(01)00116-2 11782314

[B154] Scorrano L. Oakes S. A. Opferman J. T. Cheng E. H. Sorcinelli M. D. Pozzan T. (2003). BAX and BAK regulation of endoplasmic reticulum Ca^2+^: a control point for apoptosis. Science 300, 135 10.1126/science.1081208 12624178

[B155] Scorrano L. Penzo D. Petronilli V. Pagano F. Bernardi P. (2001). Arachidonic acid causes cell death through the mitochondrial permeability transition. Implications for tumor necrosis factor-α apoptotic signaling. J. Biol. Chem. 276, 12035–12040 10.1074/jbc.M010603200 11134037

[B156] Shulga N. Pastorino J. G. (2010). Ethanol sensitizes mitochondria to the permeability transition by inhibiting deacetylation of cyclophilin-D mediated by sirtuin-3. J. Cell Sci. 123, 4117–4127 10.1242/jcs.073502 21062897 PMC2987442

[B157] Siemen D. Ziemer M. (2013). What is the nature of the mitochondrial permeability transition pore and what is it not? IUBMB Life 65, 255–262 10.1002/iub.1130 23341030

[B158] Šileikyte J. Petronilli V. Zulian A. Dabbeni-Sala F. Tognon G. Nikolov P. (2011). Regulation of the inner membrane mitochondrial permeability transition by the outer membrane translocator protein (peripheral benzodiazepine receptor). J. Biol. Chem. 286, 1046–1053 10.1074/jbc.M110.172486 21062740 PMC3020711

[B159] Siliprandi D. Siliprandi N. Toninello A. (1983). On the relationship between calcium and phosphate transport, transmembrane potential and acetoacetate-induced oxidation of pyridine nucleotides in rat liver mitochondria. Eur. J. Biochem. 130, 173–175 10.1111/j.1432-1033.1983.tb07133.x 6825686

[B160] Sinha Roy S. Madesh M. Davies E. Antonsson B. Danial N. Hajnoczky G. (2009). Bad targets the permeability transition pore independent of Bax or Bak to switch between Ca^2+^-dependent cell survival and death. Mol. Cell 33, 377–388 10.1016/j.molcel.2009.01.018 19217411 PMC2662194

[B161] Sokolove P. M. Shinaberry R. G. (1988). Na^+^-independent release of Ca^2+^ from rat heart mitochondria. Induction by adriamycin aglycone. Biochem. Pharmacol. 37, 803–812 3345198 10.1016/0006-2952(88)90165-7

[B162] Sorgato M. C. Keller B. U. Stühmer W. (1987). Patch-clamping of the inner mitochondrial membrane reveals a voltage-dependent ion channel. Nature 330, 498–500 10.1038/330498a0 2446143

[B163] Stelzer A. C. Frazee R. W. Van Huis C. Cleary J. Opipari A. W. Jr. Glick G. D. (2010). NMR studies of an immunomodulatory benzodiazepine binding to its molecular target on the mitochondrial F1F0-ATPase. Biopolymers 93, 85–92 10.1002/bip.21306 19768783

[B164] Strauss M. Hofhaus G. Schroder R. R. Kühlbrandt W. (2008). Dimer ribbons of ATP synthase shape the inner mitochondrial membrane. EMBO J. 27, 1154–1160 10.1038/emboj.2008.35 18323778 PMC2323265

[B165] Sultan A. Sokolove P. M. (2001). Free fatty acid effects on mitochondrial permeability: an overview. Arch. Biochem. Biophys. 386, 52–61 10.1006/abbi.2000.2195 11361000

[B166] Susin S. A. Zamzami N. Castedo M. Hirsch T. Marchetti P. Macho A. (1996). Bcl-2 inhibits the mitochondrial release of an apoptogenic protease. J. Exp. Med. 184, 1331–1341 8879205 10.1084/jem.184.4.1331 PMC2192812

[B167] Szabó I. Bernardi P. Zoratti M. (1992). Modulation of the mitochondrial megachannel by divalent cations and protons. J. Biol. Chem. 267, 2940–2946 1371109

[B168] Szabó I. Bock J. Grassme H. Soddemann M. Wilker B. Lang F. (2008). Mitochondrial potassium channel Kv1.3 mediates Bax-induced apoptosis in lymphocytes. Proc. Natl. Acad. Sci. U.S.A. 105, 14861–14866 10.1073/pnas.0804236105 18818304 PMC2567458

[B169] Szabó I. Soddemann M. Leanza L. Zoratti M. Gulbins E. (2011). Single-point mutations of a lysine residue change function of Bax and Bcl-xL expressed in Bax- and Bak-less mouse embryonic fibroblasts: novel insights into the molecular mechanisms of Bax-induced apoptosis. Cell Death Differ. 18, 427–438 10.1038/cdd.2010.112 20885444 PMC3132001

[B170] Szabó I. Zoratti M. (1991). The giant channel of the inner mitochondrial membrane is inhibited by cyclosporin A. J. Biol. Chem. 266, 3376–3379 1847371

[B171] Szabó I. Zoratti M. (1992). The mitochondrial megachannel is the permeability transition pore. J. Bioenerg. Biomembr. 24, 111–117 1380498 10.1007/BF00769537

[B172] Tafani M. Karpinich N. O. Hurster K. A. Pastorino J. G. Schneider T. Russo M. A. (2002). Cytochrome c release upon Fas receptor activation depends on translocation of full-length bid and the induction of the mitochondrial permeability transition. J. Biol. Chem. 277, 10073–10082 10.1074/jbc.M111350200 11790791

[B173] Takahashi N. Hayano T. Suzuki M. (1989). Peptidyl-prolyl cis-trans isomerase is the cyclosporin A-binding protein cyclophilin. Nature 337, 473–475 10.1038/337473a0 2644542

[B174] Tapley D. F. (1956). The effect of thyroxine and other substances on the swelling of isolated rat liver mitochondria. J. Biol. Chem. 222, 325–339 13367006

[B175] Thomas D. Bron P. Weimann T. Dautant A. Giraud M. F. Paumard P. (2008). Supramolecular organization of the yeast F1Fo-ATP synthase. Biol. Cell 100, 591–601 10.1042/BC20080022 18447829

[B176] Tomasetig L. Di Pancrazio F. Harris D. A. Mavelli I. Lippe G. (2002). Dimerization of F0F1ATP synthase from bovine heart is independent from the binding of the inhibitor protein IF1. Biochim. Biophys. Acta 1556, 133–141 10.1016/S0005-2728(02)00344-4 12460670

[B177] Uribe-Carvajal S. Luévano-Martìnez L. A. Guerrero-Castillo S. Cabrera-Orefice A. Corona-de-la-Peña N. A. Gutiérrez-Aguilar M. (2011). Mitochondrial unselective channels throughout the eukaryotic domain. Mitochondrion 11, 382–390 10.1016/j.mito.2011.02.004 21385626

[B178] Vaseva A. V. Marchenko N. D. Ji K. Tsirka S. E. Holzmann S. Moll U. M. (2012). p53 opens the mitochondrial permeability transition pore to trigger necrosis. Cell 149, 1536–1548 10.1016/j.cell.2012.05.014 22726440 PMC3383624

[B179] Vercesi A. E. (1984). Dissociation of NAD(P)^+^-stimulated mitochondrial Ca^2+^ efflux from swelling and membrane damage. Arch. Biochem. Biophys. 232, 86–91 10.1016/0003-9861(84)90523-X 6742863

[B180] Verma A. Nye J. S. Snyder S. H. (1987). Porphyrins are endogenous ligands for the mitochondrial (peripheral-type) benzodiazepine receptor. Proc. Natl. Acad. Sci. U.S.A. 84, 2256–2260 3031675 10.1073/pnas.84.8.2256 PMC304628

[B181] Vinogradov A. Scarpa A. Chance B. (1972). Calcium and pyridine nucleotide interaction in mitochondrial membranes. Arch. Biochem. Biophys. 152, 646–654 10.1016/0003-9861(72)90261-5 4344129

[B182] von Stockum S. Basso E. Petronilli V. Sabatelli P. Forte M. A. Bernardi P. (2011). Properties of Ca^2+^ transport in mitochondria of *Drosophila melanogaster*. J. Biol. Chem. 286, 41163–41170 10.1074/jbc.M111.268375 21984833 PMC3308830

[B183] Walsh C. T. Zydowsky L. D. McKeon F. D. (1992). Cyclosporin, A, the cyclophilin class of peptidylprolyl isomerases, and blockade of T cell signal transduction. J. Biol. Chem. 267, 13115–13118 1618811

[B184] Walter L. Miyoshi H. Leverve X. Bernardi P. Fontaine E. (2002). Regulation of the mitochondrial permeability transition pore by ubiquinone analogs. A progress report. Free Radic. Res. 36, 405–412 12069104 10.1080/10715760290021252

[B185] Walter L. Nogueira V. Leverve X. Bernardi P. Fontaine E. (2000). Three classes of ubiquinone analogs regulate the mitochondrial permeability transition pore through a common site. J. Biol. Chem. 275, 29521–29527 10.1074/jbc.M004128200 10889201

[B186] Wang J. Q. Chen Q. Wang X. Wang Q. C. Wang Y. Cheng H. P. (2013). Dysregulation of mitochondrial calcium signaling and superoxide flashes cause mitochondrial genomic DNA damage in Huntington disease. J. Biol. Chem. 288, 3070–3084 10.1074/jbc.M112.407726 23250749 PMC3561531

[B187] Wang P. Heitman J. (2005). The cyclophilins. Genome Biol. 6, 226.1–226.6 10.1186/gb-2005-6-7-226 15998457 PMC1175980

[B188] Wang W. Fang H. Groom L. Cheng A. Zhang W. Liu J. (2008). Superoxide flashes in single mitochondria. Cell 134, 279–290 10.1016/j.cell.2008.06.017 18662543 PMC2547996

[B189] Wei-Lapierre L. Gong G. Gerstner B. J. Ducreux S. Yule D. I. Pouvreau S. (2013). Respective contribution of mitochondrial superoxide and pH to Mt-cpYFP flash activity. J. Biol. Chem. 288, 10567–10577 10.1074/jbc.M113.455709 23457298 PMC3624438

[B190] Wojtczak L. Lehninger A. L. (1961). Formation and disappearance of an endogenous uncoupling factor during swelling and contraction of mitochondria. Biochim. Biophys. Acta 51, 442–456 14007889 10.1016/0006-3002(61)90600-x

[B191] Woodfield K. Y. Price N. T. Halestrap A. P. (1997). cDNA cloning of rat mitochondrial cyclophilin. Biochim. Biophys. Acta 1351, 27–30 10.1016/S0167-4781(97)00017-1 9116040

[B192] Yamada A. Yamamoto T. Yoshimura Y. Gouda S. Kawashima S. Yamazaki N. (2009). Ca^2+^-induced permeability transition can be observed even in yeast mitochondria under optimized experimental conditions. Biochim. Biophys. Acta 1787, 1486–1491 10.1016/j.bbabio.2009.07.001 19616504

[B193] Youle R. J. Narendra D. P. (2011). Mechanisms of mitophagy. Nat. Rev. Mol. Cell. Biol. 12, 9–14 10.1038/nrm3028 21179058 PMC4780047

[B194] Zamzami N. Kroemer G. (2001). The mitochondrion in apoptosis: how Pandora's box opens. Nat. Rev. Mol. Cell. Biol. 2, 67–71 10.1038/35048073 11413468

[B195] Zborowski J. Wojtczak L. (1963). Induction of swelling of liver mitochondria by fatty acids of various chain length. Biochim. Biophys. Acta 70, 596–598 14085946 10.1016/0006-3002(63)90799-6

[B196] Zoeteweij J. P. van de Water B. de Bont H. J. Mulder G. J. Nagelkerke J. F. (1993). Calcium-induced cytotoxicity in hepatocytes after exposure to extracellular ATP is dependent on inorganic phosphate. Effects on mitochondrial calcium. J. Biol. Chem. 268, 3384–3388 8429014

[B197] Zoratti M. Szabó I. De Marchi U. (2005). Mitochondrial permeability transitions: how many doors to the house? Biochim. Biophys. Acta 1706, 40–52 10.1016/j.bbabio.2004.10.006 15620364

[B198] Zulian A. Petronilli V. Bova S. Dabbeni-Sala F. Cargnelli G. Cavalli M. (2007). Assessing the molecular basis for rat-selective induction of the mitochondrial permeability transition by norbormide. Biochim. Biophys. Acta 1767, 980–988 10.1016/j.bbabio.2007.04.002 17509521

